# Dynamic feature of infrapatellar fat pad during walking in patients with knee osteoarthritis

**DOI:** 10.1007/s10396-025-01518-3

**Published:** 2025-02-22

**Authors:** Miharu Sugimoto, Yosuke Ishii, Yuko Nakashima, Goki Kamei, Akinori Nekomoto, Takato Hashizume, Riko Okinaka, Kohei Matsumura, Makoto Takahashi, Nobuo Adachi

**Affiliations:** 1 Department of Biomechanics, Graduate School of Biomedical and Health Sciences, Hiroshima University, 1-2-3 Kasumi, Minami-ku, Hiroshima, 734-8553 Japan; 2 Department of Orthopaedic Surgery, Chugoku Rosai Hospital, Hiroshima, Japan; 3 Department of Orthopaedic Surgery, Graduate School of Biomedical and Health Sciences, Hiroshima University, Hiroshima, Japan; 4 Department of Sports Medical Center, Hiroshima University Hospital, Hiroshima, Japan

**Keywords:** Knee osteoarthritis, Walking, Dynamic evaluation, Infrapatellar fat pad, Morphological change

## Abstract

**Purpose:**

The infrapatellar fat pad (IFP) absorbs mechanical stress in the knee joint owing to flexible morphological changes. The IFP is a key factor in knee osteoarthritis (OA); however, its dynamic feature during walking remains unknown. This study aimed to investigate whether the morphological changes in the IFP during walking involve specific features for patients with knee OA.

**Methods:**

Twelve patients with knee OA, 12 healthy young volunteers, and 12 healthy elderly volunteers were recruited. The IFP was evaluated using ultrasonography, and dynamics were recorded in video mode during walking. The IFP value was identified as the thickness between the patellar tendon and proximal tibial line. The morphological change in the IFP (ΔIFP) was shown as the difference in IFP value between maximum and at initial contact on the waveform. Kinematics and kinetics data were evaluated using a three-dimensional motion analysis system synchronized with ultrasonography, and the knee flexion angle and its moment in the stance phase were evaluated.

**Results:**

In the patients with knee OA, the ΔIFP was lower than that in healthy volunteers, but there was no difference between control groups (knee OA: 1.4 ± 0.3 mm, elderly control: 1.8 ± 0.2 mm, young control: 2.1 ± 0.5 mm, p < 0.05). In all the groups, there was no significant correlation between the IFP values and kinetic parameters, including the range of knee flexion angles and gait speed.

**Conclusion:**

Insufficient morphological changes in the IFP during walking could be a feature of knee OA.

## Introduction

Knee osteoarthritis (OA) is a degenerative disease induced by multiple factors, especially mechanical factors such as loading stress on the joint [1]. It often leads to joint movement limitation and pain during walking, resulting in poor physical activities of daily living [2, 3]. Therefore, it is necessary to sensitively observe the OA-associated mechanical stress and develop an appropriate approach for the prevention of worsening clinical conditions.

Recently, it has been suggested that the pathology of knee OA involves not only the articular cartilage but also the entire joint organ, including the meniscus, periarticular muscle, synovium, and local fat pads [1, 4]. One of these is the infrapatellar fat pad (IFP), located in the frontal compartment of the knee joint. The IFP absorbs the mechanical stress on the frontal region of the knee through flexible morphological changes [5–7]. On the other hand, patients with knee OA often undergo poor shape change and histological changes in the IFP such as inflammation, swelling, and fibrosis [8, 9]. These pathological conditions raise concerns about the inability to distribute mechanical stress and knee pain [9–13]. Importantly, patients experience anterior knee pain while walking, which may reflect excessive mechanical stress in a localized area that occurs during daily life. Several previous studies, using magnetic resonance imaging (MRI) or ultrasonography, evaluated the morphological changes in the IFP according to knee movement and load only under static conditions [14–16]. However, considering that mechanical stress under static conditions is smaller than that under dynamic conditions [17], these evaluations may not reflect IFP dynamics during daily life.

Recently, dynamic changes in the medial meniscus during walking have been captured using dynamic ultrasound evaluation, revealing the specific features in patients with knee OA [18–20]. Moreover, in healthy volunteers, the feature of morphological changes in the IFP during walking have been shown [21]. However, IFP dynamics in patients with knee OA during walking have not been investigated. Dynamic ultrasound evaluation has the possibility to detect distinctive morphological changes in the IFP observed during walking in patients with knee OA, which could be helpful in identifying the unique characteristics reflected in knee OA pathology during daily life.

This study aimed to investigate the differences in morphological changes in the IFP during walking between patients with knee OA and healthy volunteers using dynamic ultrasound evaluation to reveal the IFP dynamics during walking in patients with knee OA. We hypothesized that patients with knee OA experience less morphological change in the IFP compared to healthy volunteers.

## Material and methods

### Participants

This cross-sectional study recruited 12 unilateral or bilateral knee OA patients (OA group: mean age 65.0 ± 8.8 years, males n = 7), and 12 young and 12 elderly asymptomatic volunteers as the control groups (elderly group: mean age 59.8 ± 8.0 years, males n = 5; young group: mean age 23.0 ± 1.0 years, males n = 4). All the participants were able to perform daily activities and walk smoothly without support. The exclusion criteria included traumatic knee injuries, surgical treatment for knee problems, and a history of neuromuscular disorders. This study was approved by the Ethical Committee for Epidemiology of Hiroshima University (E-2498-2), and all participants provided appropriate informed consent.

### Radiological assessments

A radiographic examination was performed in all patients with knee OA to assess the severity and varus alignment. Kellgren-Laurence grade (KL) was used to assess severity, and femorotibial angle (FTA) was used to measure varus knee alignment. All evaluations were performed by a single orthopedic surgeon. If the patient had bilateral medial knee OA, the more severe and painful knee was considered the affected side in this study.

### Gait analyses

A three-dimensional motion analysis system (Vicon Motion Systems, Oxford, UK) and 16 cameras (Vicon Motion Systems, Oxford, UK) were used to obtain kinematical data. Eight force platforms (AMTI, Watertown, Mass) were systematically synchronized with cameras, and kinetic data were measured. The cameras and force platforms had sampling rates of 100 and 1000 Hz, respectively. Before initiating the measurements, the cameras were masked and calibrated to reduce tracking errors and identify the axes of each joint in spatial coordinates. Subsequently, the examiner attached 16 reflective markers to the participant’s bodies at their anatomical landmarks, adopting the standard Plug-in-Gait lower body model (Vicon^®^ Peak, Vicon Motion Systems).

For three trials, the participants were required to walk 5 m straight at a comfortable, self-selected speed. Raw data were filtered with a Butterworth 4th-order filter, with a cutoff frequency of 6 Hz based on the Plug-in-Gait software (Vicon^®^ Peak, Vicon Motion Systems). The stance phase, which is the period from initial contact to toe-off on the ipsilateral leg, was the subject of the analysis section, and normalized to 100 data points. These gait events were recognized using a threshold of vertical ground reaction force (GRF) 20 N. The knee flexion range of motion and its moment were obtained from the spatial coordinates and GRF signal using Nexus 2.14.0 (Vicon Motion Systems). The knee flexion angle range was calculated from the difference in the angle at heel contact and the first peak in the early stance phase. Biomechanical data, including maximum and impulse of knee flexion moment and GRF peaks, were calculated, and knee flexion moment parameters were normalized to body weight. In addition, spatiotemporal parameters such as gait speed were collected by calculating the information from their heel markers.

### Evaluation of IFP behavior

**Fig. 1:**
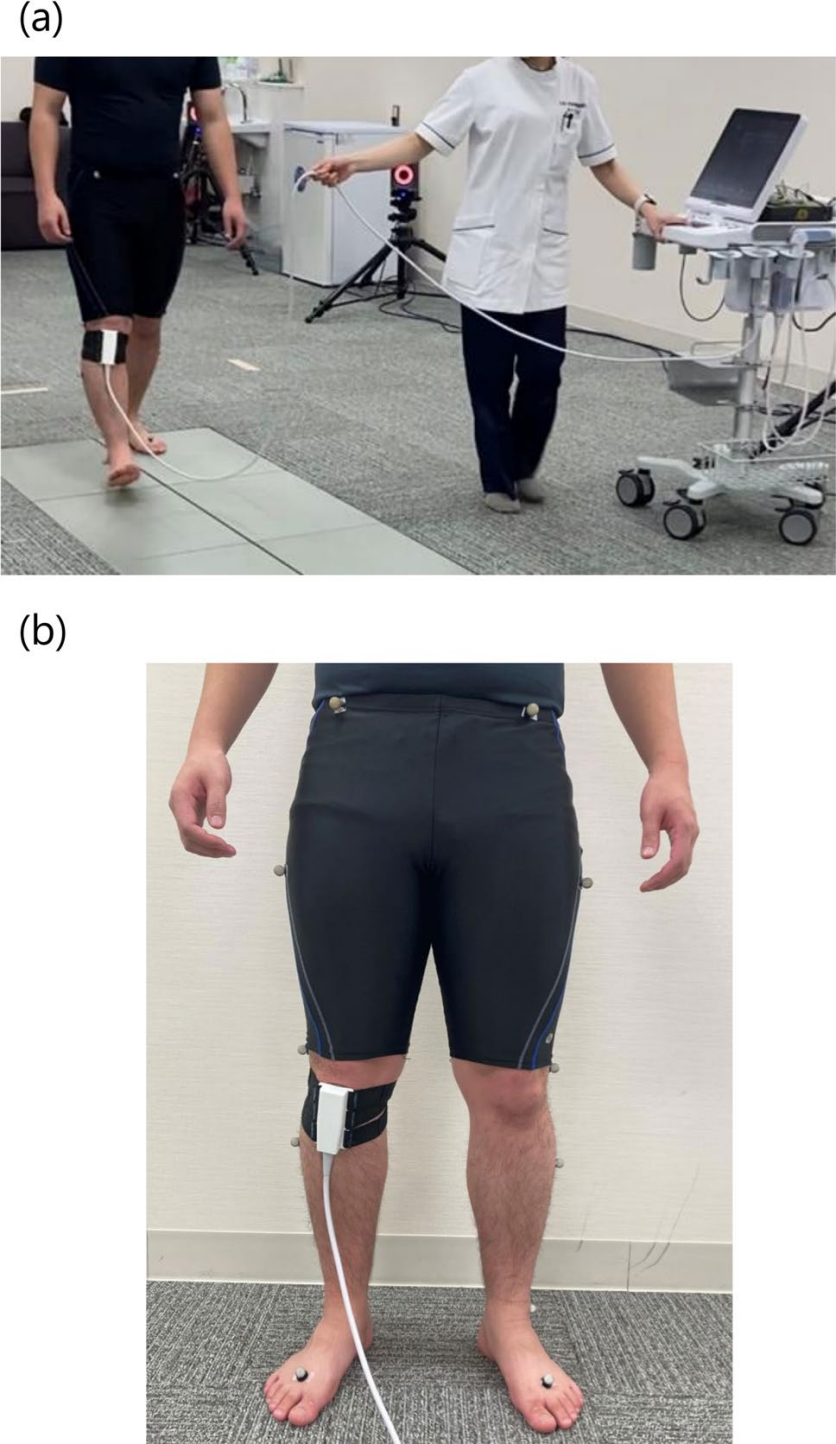
Snapshot of motion analysis during walking (**a**) and a transducer placed at the frontal knee surface between tibial tuberosity and under the patella (**b**)

**Fig. 2:**
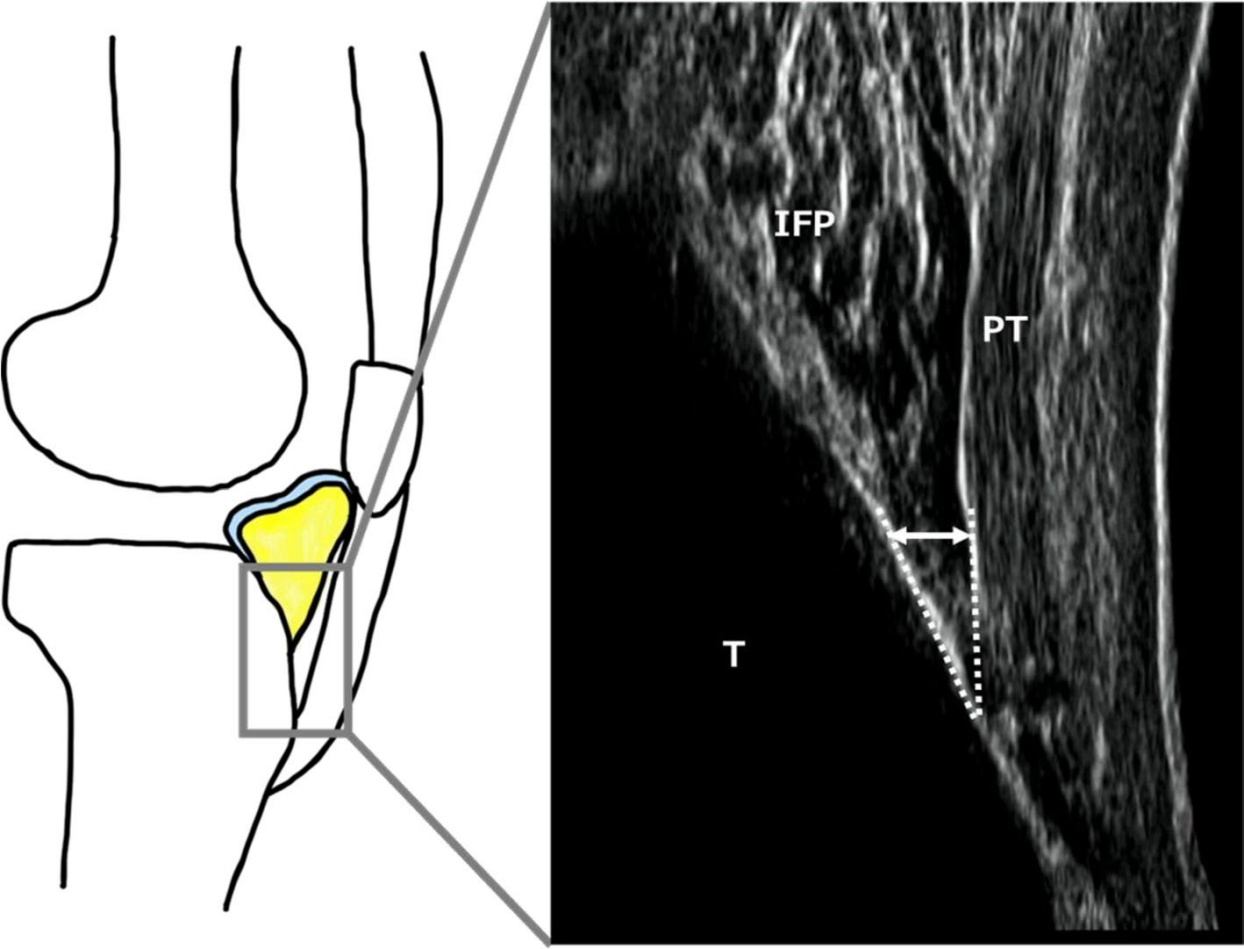
Representative image of the IFP. Two dotted lines show the undersurface of the PT and tibia cortex. The arrow shows the thickness of the IFP indicated by a perpendicular line drawn from the tendon to the cortex point, 10 mm proximal to the tendon insertion. IFP, infrapatellar fat pad; PT, patella tendon; T, Tibia

IFP data during comfortable 5-m walks were obtained using ultrasonography (SNiBLE; KONICA MINOLTA, Japan) with a new prototype linear-array transducer (3–11 MHz) (KONICA MINOLTA, Japan) (Fig. 1a). According to previous studies on the IFP [22], the transducer was placed at the frontal knee surface between tibial tuberosity and under the patella using a flexible belt that can adopt several knee flexion angles (Fig. 1b). In terms of technical aspects, the examiner first confirmed whether the IFP was located between the patellar tendon (PT) and proximal tibia. The participants were then asked to contract their quadricep muscles to observe a straight PT. Through these processes, a clear ultrasound image was obtained (Fig. 2).

**Fig. 3:**
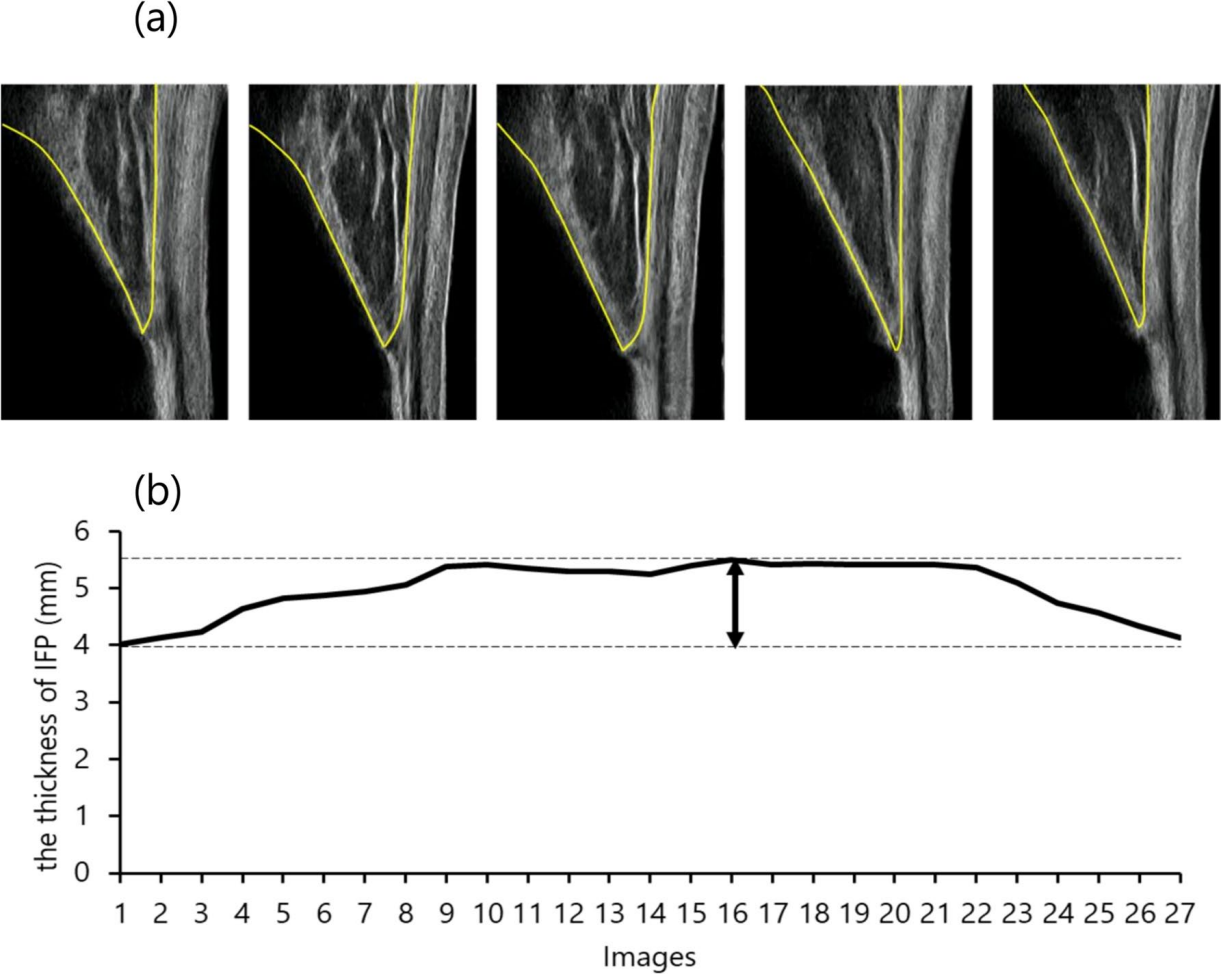
Representative images of IFP dynamics during the stance phase of the single gait cycle (**a**). The yellow lines show the region of the IFP. The waveform of IFP thickness during the stance phase (**b**); dashed lines indicate the maximum value and the value at initial contact. ∆ is indicated by arrow between dashed lines. IFP, infrapatellar fat pad

Ultrasonography was synchronized with a motion analysis system, and the IFP, kinematics, and kinetic data were simultaneously obtained. The IFP was evaluated in the stance phase on the single gait cycle and recorded in the video mode with a 30 Hz sample rate. Approximately 20 ultrasound images were obtained in a single step. Following a previous study [22], the IFP thickness was defined as a vertical line to the undersurface of the PT from the tibial cortex point, which was located 10 mm proximal to the PT insertion. It was calculated for each image using the Kinovea software (v0.827, Kinovea open-source project), and the IFP waveform was composed of sequential IFP values (Fig. 3). Moreover, the waveform was normalized to 100 data points to compare different numbers of images. The difference between the IFP thickness at initial contact and maximum in the stance phase was calculated as morphological changes in IFP defined as ΔIFP. Eventually, the representative value, which was determined by averaging three random steps, was used in statistical analyses. We also calculated the thickness of the IFP in the supine and standing positions.

To demonstrate the reliability of the IFP thickness, a preliminary study was conducted by two examiners and included repeated retest trials using eight knees of eight healthy volunteers. The intraclass correlation coefficient (ICC) was used to analyze the intra- and inter-rater reliabilities. Regarding the maximum IFP thickness during walking, ICC (1,3) and ICC (2,3) were 0.942 and 0.890, respectively. Regarding the minimum IFP thickness during walking, ICC (1,3) and ICC (2,3) were 0.930 and 0.737, respectively. Regarding ΔIFP during walking, ICC (1,3) and ICC (2,3) were 0.857 and 0.602, respectively [21].

### Statistical analyses

Demographic, IFP, and kinematic and kinetic parameters were analyzed using SPSS Version 23 (Released 2015; IBM Corp., Armonk, NY, USA). For each data point, normality was confirmed using the Shapiro–Wilk assay. To compare groups, one-way analysis of variance or the Kruskal–Wallis test was performed, and the Tukey test or Steel–Dwass test was used for multiple comparisons. In addition, correlation analysis using Pearson or Spearman were conducted to investigate the relationship between ΔIFP and gait parameters, including the knee angle and moment. The significance level was set at p < 0.05.

The pos-hoc analyses demonstrated using G*power 3.1.9.7 calculated the significant power to detect the ΔIFP difference between patients with knee OA and healthy elderly volunteers. Effect size and power were 0.86 and 99%, respectively.

## Results

### Demographic data of participants

Demographic data of participants are presented in Table 1. All patients with knee OA had mild to moderately severe OA and their FTA was 179.1 ± 2.9° (mean ± standard deviation). There was no significant correlation between ΔIFP and KL of the tibiofemoral and patellofemoral joints, respectively (TF: r = 0.03, p = 0.94; PF: r = -0.15, p = 0.67). The age of young volunteers was significantly lower than that of patients with knee OA and elderly volunteers. There were no significant differences in other parameters among the groups.

**Table 1:** Demographic data of the participant s

	Knee OA	Elderly control	Young control
N/knees	11/11	12/12	12/12
Sex (M:F)	7:4	5:7	4:8
Age (years)	65.0 ± 8.8	59.8 ± 8.0	23.0 ± 1.0*
Height (cm)	161.1 ± 8.6	167.2 ± 8.7	163.8 ± 5.5
BMI (kg/m^2^)	24.1 ± 1.9	23.9 ± 3.9	21.1 ± 2.2
FTA (°)	179.1 ± 2.9	–	–
KL-TF (I, II, III, IV)	0, 6, 4, 1	–	–
KL-PF (I, II, III, IV)	4, 4, 3, 0	–	–

Values represent mean ± standard deviation

BMI, body mass index; FTA, femorotibial angle; KL, Kellgren-Laurence grade; TF, tibiofemoral joint; PF, patellofemoral joint

*Difference between knee OA group and each control group using the post-hoc test (p < 0.05)

### Biomechanical data during walking

The range of knee flexion angle and gait speed in the knee OA group were significantly smaller than in the control groups. However, no significant variations were noted between the groups in terms of other parameters such as the peaks and impulse of knee flexion moment and GRF (Table 2).

**Table 2:** Biomechanical data during walking between the groups

	Knee OA	Elderly	Young
Range of knee flexion angle (°)	7.4 ± 1.8	10.9 ± 2.5*	9.3 ± 2.0
Maximum KFM (Nm/kg)	0.48 ± 0.2	0.69 ± 0.2	0.47 ± 0.2
Impulse KFM (Nm s/kg)	0.19 ± 0.2	0.21 ± 0.1	0.12 ± 0.1
GRF (N)	614.8 ± 97.8	679.3 ± 106.3	592.4 ± 70.6
Gait speed (m/s)	0.76 ± 0.2	1.00 ± 0.1*	0.96 ± 0.1*

Values represent mean ± standard deviation

KFM, knee flexion moment; GRF, ground reaction force

*Difference between knee OA group and each control group using the post-hoc test (p < 0.05)

### Waveforms of morphological changes in the IFP during the stance phase

**Fig. 4:**
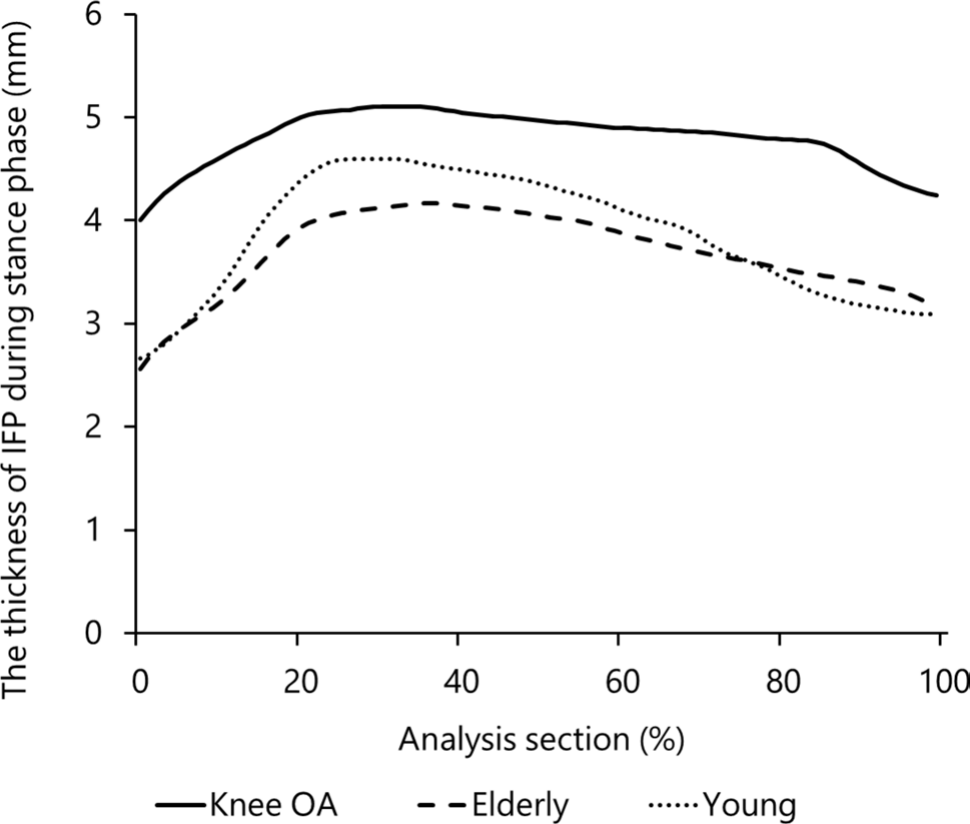
The thickness of the IFP during the stance phase is shown as a waveform. This waveform was normalized to 100 data points to compare the different images. Solid, coarse, and subtle lines represent the knee OA group, elderly control group, and young control group, respectively. IFP, infrapatellar fat pad

In all the groups, the IFP moved distally during the early stance phase and proximally during the late stance phase. In elderly and young volunteers, the thickness of the IFP peaked during the early stance phase and subsequently gradually decreased. In contrast, IFP thickness in patients with knee OA did not change considerably, showing an irregular waveform (Fig. 4).

**Fig. 5:**
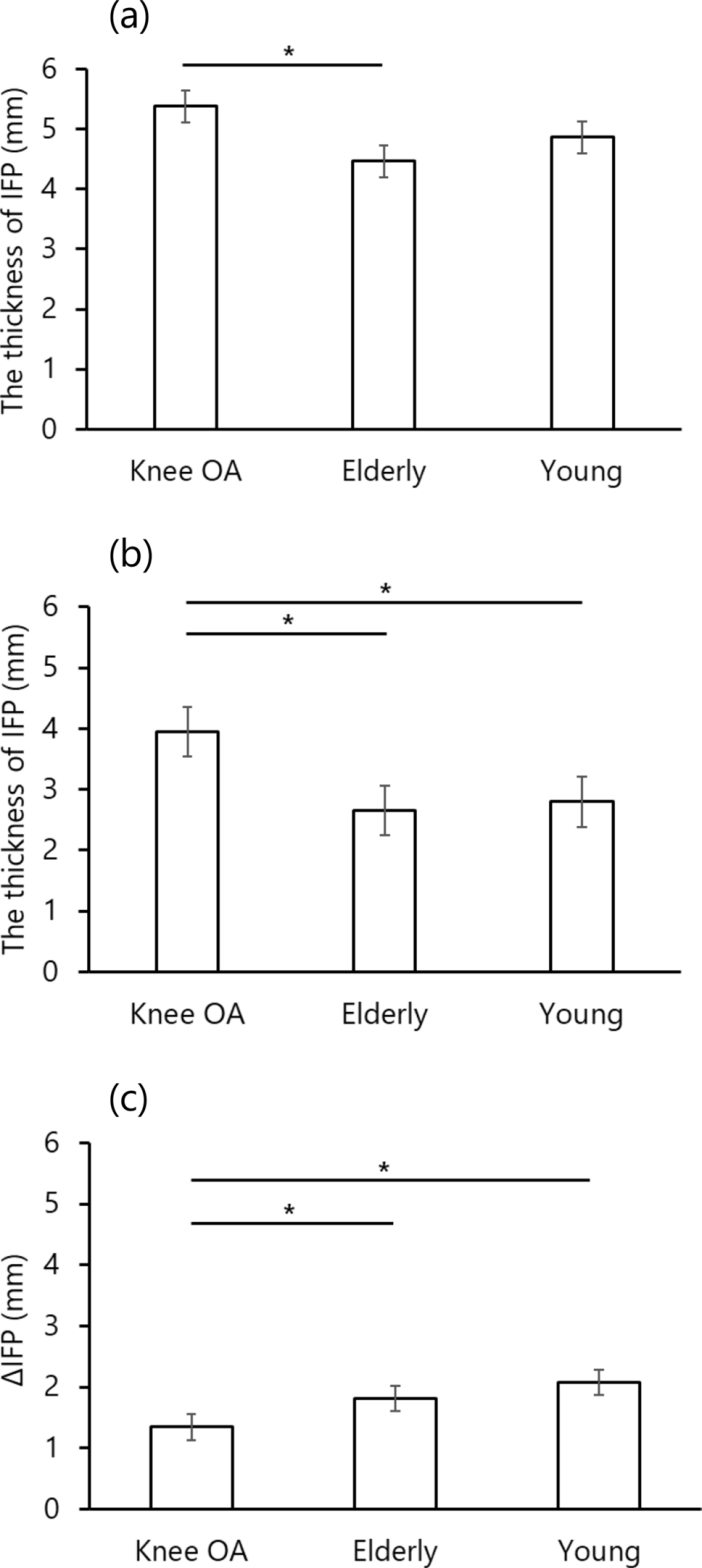
Comparison of IFP values between the groups. **a**–**c** Show the maximum, minimum, and ∆ of IFP thickness during the stance phase, respectively. Values are represented as mean ± standard deviation. *Represents a significant difference between groups or conditions (p < 0.05). IFP, infrapatellar fat pad; ∆IFP, morphological changes in IFP

The maximum IFP value was higher in the knee OA group than in the elderly control group, and the minimum IFP value was higher in the knee OA group than in both control groups. ΔIFP was lower in the knee OA group than in both control groups (Table 3, Fig. 5).

**Table 3:** IFP values during walking

	Knee OA	Elderly	Young
IFP maximum (mm)	5.4 ± 0.9	4.4 ± 0.9*	4.8 ± 0.7
IFP minimum (mm)	4.0 ± 1.1	2.6 ± 0.9*	2.7 ± 0.7*
ΔIFP (mm)	1.4 ± 0.3	1.8 ± 0.2*	2.1 ± 0.5*

Δ, amount of variance in the value of the thickness of IFP from maximum to minimum

Values represent mean ± standard deviation

IFP, infrapatellar fat pad

*Difference between knee OA group and each control group using the post-hoc test (p < 0.05)

No significant differences were observed between the groups in terms of the thickness of the IFP under supine and standing conditions (Table 4).

**Table 4:** IFP values under supine and standing conditions

	Knee OA	Elderly	Young
IFP supine (mm)	5.0 ± 0.8	5.0 ± 0.7	5.4 ± 0.9
IFP standing (mm)	4.7 ± 0.8	4.8 ± 0.9	5.0 ± 0.6
ΔIFP (mm)	0.3 ± 0.8	0.2 ± 1.3	0.4 ± 1.0

Δ, amount of variance in the value of the thickness of IFP under supine and standing conditions

Values represent mean ± standard deviation

IFP, infrapatellar fat pad

*Difference between knee OA group and each control group using the post-hoc test (p < 0.05)

### Correlation between ΔIFP and kinematic or kinetic parameters

No significant correlation was found in any group between the range of knee flexion angle and ΔIFP (knee OA: r = − 0.42, p = 0.20; elderly volunteers: r = 0.10, p = 0.75; young volunteers: r = 0.071, p = 0.83), nor between gait speed and ΔIFP (knee OA: r = − 0.08, p = 0.80; elderly volunteers: r = − 0.074, p = 0.94; young volunteers: r = 0.17, p = 0.60).

## Discussion

Our data revealed that morphological changes in the IFP during walking were smaller in the knee OA group compared to those in both the elderly and young control groups. The difference in the dynamic feature of the IFP during walking between patients with knee OA and healthy volunteers supports our hypothesis.

However, no significant difference was observed between the knee OA group and both control groups in terms of biomechanical factors such as knee flexion moment and GRF. The gait speed in healthy volunteers was significantly higher than that in the knee OA group, which was similar to the trend observed in previous studies [23, 24]. Generally, patients with knee OA experience greater mechanical stress than healthy volunteers [25, 26]. However, gait parameters, particularly moments that depend on gait speed and pain, often mask knee OA features [24, 27]. Therefore, gait analyses using only biomechanical data raise concerns about the inconsistency of the mechano-pathology in patients with knee OA. On the other hand, in the knee OA group, the IFP parameters, including ΔIFP, were evidently different from those of the control groups. In particular, the insufficient morphological changes mean dysfunction of absorbing the mechanical stress [8, 15]. Our data were in line with previous studies that showed less dynamics of the IFP under static conditions for patients with knee OA [8] and postoperative symptomatic ACL knees [16]. Therefore, several previous studies and our data could explain our observation that the dynamics of the IFP during walking, which assume the IFP works more under mechanical stress, could describe the knee OA.

Our results showed that there was no significant difference between elderly and young volunteers in the amount of morphological change in the IFP during walking, which signified that the ability of morphological change in the IFP is not affected by aging itself. Knee OA is a multifactorial condition with many risk factors including aging, joint injury, obesity, and abnormal mechanics [28]. Degeneration of the articular cartilage is involved in the development and progression of knee OA [29]. However, a previous study reported that aging does not directly cause knee OA but rather increases the vulnerability of the tissue to degenerate and decreases the ability of joint tissue to defend against disease progression [30]. Knee OA has also been reported in young people [31]. Therefore, our data and those of previous studies emphasize that aging is a risk factor for knee OA, but it is not an indicator to distinguish between knee OA and healthy conditions.

The results of this study demonstrated that morphological changes in the IFP might be related to knee OA pathology. Prior investigations focused on the IFP only under static conditions, such as supine or standing positions [15, 22], and therefore IFP dynamics might have been underestimated. In this study, although there was no significant difference between the groups in terms of the thickness of the IFP in the supine and standing positions, the amount of morphological change in the IFP in patients with knee OA during walking, i.e., the ability to change its shape, decreased compared to that in healthy volunteers. This is the first study to focus on the dynamic features of the IFP in patients with knee OA during walking and could be the first step in detecting the disease. Thus, our findings emphasize the clinical reaction of the IFP under mechanical stress and could provide clinicians with valuable insights into the pathology of knee OA.

This study had several limitations. This study had a small sample size; therefore, the findings may not sufficiently reflect the overall trend in patients with knee OA. The IFP data in this study were measured only at the distal part of the IFP and no MRI images were available. Therefore, if we had focused on the dynamics of the entire IFP and classified patients with knee OA according to their severity levels and whether they had IFP inflammation or fibrosis, we might have been able to provide more detailed information. In terms of morphological changes, the IFP changes shape with muscle activity, but we were not able to mention this. Moreover, the factors that affect the morphological change in IFP during walking were not fully investigated. In future studies, we need to prepare a sufficient dataset and recruit patients based on their severity. Along with focusing on kinematic and kinetic parameters, it requires collecting histological evaluations, entire dynamic assessments, and muscle activity. It is also crucial to make clear how clinical symptoms relate to IFP dynamics.

## Conclusion

Insufficient morphological changes in IFP during walking could be a feature of knee OA.

## Data Availability

The datasets generated and/or analyzed during the current study are available from the corresponding author upon reasonable request.
